# Effect of stump-preserving arthroscopic reconstruction or stump-eliminating arthroscopic reconstruction combined with exercise rehabilitation therapy on knee functional recovery in patients with anterior cruciate ligament injuries

**DOI:** 10.1308/rcsann.2025.0056

**Published:** 2025-07-29

**Authors:** S Li, S Tang, B Zhu, Y Zhong, X Ren

**Affiliations:** Ankang Hospital of Traditional Chinese Medicine, China

**Keywords:** Anterior cruciate ligament injury, Arthroscopy, Stump-preserving arthroscopic reconstruction, Stump-eliminating arthroscopic reconstruction, Exercise rehabilitation therapy, Knee function, Knee mobility, Proprioception

## Abstract

**Introduction:**

The aim of this study was to evaluate the effect of stump-preserving arthroscopic anterior cruciate ligament (ACL) reconstruction (ACLR) combined with exercise rehabilitation therapy on knee functional recovery in patients with ACL injuries.

**Methods:**

Patients with ACL injuries (*n* = 120) were randomly divided into an observation group (60 patients; stump-preserving ACLR) or a control group (60 patients; stump-eliminating ACLR) using the randomised numerical table method. Both groups underwent a 12-week postoperative exercise rehabilitation treatment. Pain, swelling and range of motion (ROM) were recorded. Before reconstruction, and at 3, 6 and 12 months after reconstruction, proprioception was assessed by threshold to detection of passive motion (TTDPM) and passive angle regeneration test, and knee function was assessed using Lysholm and International Knee Documentation Committee (IKDC) function scores. Postoperative complications were recorded in both groups.

**Results:**

After ACLR, in both groups, pain and swelling were reduced, the ROM of knee flexion, extension, internal rotation and external rotation increased, the TTDPM and passive angle regeneration test results were reduced, and the Lysholm and IKDC function scores were increased. More significant improvements were seen in the observation group. The incidence of postoperative complications in the observation group was 13.33% (8 of 60), less than the 15.00% (9 of 60) in the control group (*p* > 0.05).

**Conclusions:**

Arthroscopic stump-preserving ACLR combined with exercise rehabilitation therapy can significantly reduce postoperative pain and swelling in patients with ACL injuries and improve postoperative knee mobility and proprioception.

## Introduction

The anterior cruciate ligament (ACL) attaches medially to the anterior intercondylar area of the tibia, blending partially with the anterior side of the lateral meniscus.^1^ The ACL serves as a sensory organ that provides proprioceptive feedback and initiates muscular reflexes that protect and stabilise the body. ACL injuries are common knee joint injuries that can affect the range of motion (ROM) and stability of the knee joint, damage the meniscus and articular cartilage, and even impair proprioceptive function.^2^ There is currently evidence that the injured ligament has self-healing properties; however, the ACL is surrounded by joints, and connecting the two ends can be difficult.

ACL reconstruction (ACLR) is a commonly performed surgical procedure. Ligament reconstruction can restore the greatest degree of continuity to the ligament, aid in the recovery of motor function in the knee and reduce the risk of secondary meniscus damage in the long run.^3^ The ligament stump is generally thoroughly eliminated to expose the visual field and more accurately determine the location of the bone marrow canal. There has, however, been an increase in interest in preserving ACL stumps. It may be beneficial to preserve ACL stumps to promote revascularisation, strengthen the quadriceps muscles and promote recovery of joint stability and function.^4,5^ In addition, preserving ACL stumps following ACLR has been shown to promote tendon–bone healing and rehabilitation of proprioceptive nerves.^6,7^ ACL stumps remain a matter of controversy. Therefore, higher-quality clinical evidence is required to guide clinical practice on remnant preservation vs standard techniques.

Therefore, this study was carried out to determine the efficacy of stump-preserving ACLR and stump-eliminating ACLR in combination with exercise rehabilitation therapy in patients suffering ACL injury, thereby providing a more appropriate therapy regimen for patients with ACL injury.

## Methods

### Ethical statement

This study was approved by the medical ethics committee of Ankang Hospital of Traditional Chinese Medicine. Patients signed the informed consent.

### General information

Patients with ACL injury (*n* = 120) admitted to Ankang Hospital of Traditional Chinese Medicine from January 2021 to December 2022 were selected and divided into an observation group (60 patients) or a control group (60 patients) using the random number table method. Differences in demographics between the two groups were not statistically significant (*p* > 0.05) (Table 1).

**Table 1:** Comparison of demographics between the two groups (*n* = 60)

Item	Observation group	Control group	p-value
Age, years	35.50 (29.25, 41.75)	34.50 (28.00, 42.00)	0.587
Gender, *n* (%)			0.168
Male	50 (83.33)	55 (91.67)	–
Female	10 (16.67)	5 (8.33)	–
Course of disease, *n* (%)			0.532
Within 1 week	46 (76.67)	43 (71.67)	–
Within 2 weeks	14 (23.33)	17 (28.33)	–
Cause of injury, *n* (%)			0.166
Non-contact injury	38 (63.33)	45 (75.00)	–
Contact injury	22 (36.67)	15 (25.00)	–
Location of injury, *n* (%)			0.699
Left side	19 (31.67)	21 (35.00)	–
Right side	41 (68.33)	39 (65.00)	–
Lachman test, *n* (%)			0.537
I positive	5 (8.33)	3 (5.00)	–
II positive	44 (73.33)	49 (81.67)	–
III positive	11 (18.33)	8 (13.33)	–
Pivot-shift test, *n* (%)			0.602
I positive	27 (45.00)	31 (51.67)	–
II positive	29 (48.33)	27 (45.00)	–
III positive	4 (6.67)	2 (3.33)	–

Skewed quantitative data were described by median and interquartile range [M (P25, P75)] for Mann–Whitney *U* test.

### Inclusion criteria

Patients were included in the study if they had: (1) unilateral injury within 2 weeks; (2) ACL injury diagnosed by magnetic resonance imaging, knee arthroscopy, etc.; (3) positive results for the Lachman test or pivot-shift test; (4) were aged 18 years and above; (5) simple ACL injury without posterior cruciate ligament injury, lateral collateral ligament injury, meniscus injury, tibial plateau injury, etc.; and (6) signed the informed consent.

### Exclusion criteria

Patients were not included if they had: (1) obsolete ACL injury; (2) recurrent patellar dislocation; (3) other diseases affecting the function of the knee joint; (4) serious heart, liver and kidney diseases; (5) fracture, peripheral nerve injury or multiple ligament injury; (6) neurological disorders such as Alzheimer’s disease, depression and anxiety; (7) malignant tumours, coagulation disorders or postoperative serious infections; or (8) were pregnant and lactating women.

### Arthroscopic ACLR

Both patient groups underwent unilateral epidural anaesthesia administered by the same surgical team. The patients were positioned supine, and standard parapatellar arthroscopy was conducted using the anteromedial and anterolateral approaches to thoroughly examine intra-articular structures, assess ACL injuries and manage articular cartilage. A 3cm oblique incision was made on the medial aspect of the tibial tuberosity to harvest the gracilis and semitendinosus tendons using a tendon extractor, with the option to trim the hamstring tendon according to the reconstruction needs. In the observation group, the ACL of the tibial stump was maintained at a minimum of 0.5mm intraoperatively, with partial removal of the femoral ligament stump to expose the posterior margin of the crest and cartilage of the lateral femoral condyle, as well as the femoral footprint region of the ACL. A guide was inserted through an anteroinferior approach to create the tibial channel, with the tibial guide accurately positioned at the centre of the tibial eminence footprint region. The tibial channel was drilled, and the fibres of the ligament stumps were manipulated along the stumps during this procedure, maintaining the integrity of the synovial membrane of the stumps. In the control group, the tibial stump was not preserved intraoperatively; specifically, the residual femoral and tibial ends were meticulously removed, and the corresponding femoral tunnels were created by positioning the pins at the 2:30 clock position on the left knee and at 10 o’clock on the right knee at 100° of knee flexion. In both the observation and control groups, the appropriate diameter drill bit was selected according to the size of the ligament to establish the bone tunnel. The bone tunnels were positioned using a 2-0 Achilles tendon wire as a guide, with the observation group focusing on safeguarding the stump to ensure proper wrapping of the graft by the synovial membrane and fibrous components. By contrast, the control group utilised conventional ligament-grafting methods, with the tibial tunnel secured with an extrusion screw at a 20° knee extension and the femoral tunnel immobilised using the Rigid Fix transverse nailing system. After placement of the autologous popliteal tendon, knee stability and flexion motion were tested and found to be good. In the control group, the residual ACL tissue at the tibial eminence was completely removed using an ionic knife, and the rest of the operation was the same as for the observation group.

Patients in both groups were instructed to wear external fixation braces for 1 week following reconstruction, with the knee joint maintained in full extension. Patients were permitted to bear weight with the assistance of crutches 1 week post-reconstruction, with strict instructions to avoid any knee flexion movements. Rehabilitation therapy, consisting of 12 weeks of treatment, commenced 2 weeks post-operation. The initial 6 weeks focused on passive knee flexion training, gradually increasing the ROM of the affected limb while ensuring pain was kept at a tolerable level. Contraction training involves the patient assuming a supine position and contracting the quadriceps to their maximum capacity, while also increasing hamstring exercises and holding for 5–10s. Active extension exercises entailed the patient getting out of bed for non-weight-bearing functional training, including practising straight leg raising. Balance training focused on improving balance and walking ability through activities such as walking along a straight line for 20m and repeating the exercise. In addition, one-legged standing exercises involved standing on one leg for 5–10s and alternating between legs. During the remaining 6 weeks, patients engaged in resistance training and weight-bearing exercises. Resistance training involved the use of a short-arm power bike and a standard resistance stationary bike, with knee mobility ranging from 85° to 90° and from 110° to 115°, respectively. Progressive weight-bearing training was tailored to the individual’s injury location and recovery progress, incrementally increasing the weight-bearing load until full weight-bearing was achieved.

### Observation indicators

#### Pain and swelling

Before and after ACLR, patient pain was assessed using a visual analogue scale (VAS), with a total VAS score of 0–10. The higher the score, the more severe the pain. Before and after reconstruction, the circumference of bilateral knee joints at the midpoint of the patella was measured using a tape measure in a state of knee extension and relaxation. The swelling value of the affected knee equalled the circumference of the affected knee minus the circumference of the healthy knee.

#### Range of motion

Before and after reconstruction, knee flexion and extension (0–130°), internal rotation (0–30°) and external rotation (0–40°) were measured using a bone joint protractor.

#### Recovery of proprioception

The proprioception of the affected side was evaluated by the threshold to detection of passive motion (TTDPM) and passive angle regeneration test results using an ISOMED200 isokinetic tester (D&R Company, Germany) before, and 3, 6 and 12 months after reconstruction. In the TTDPM, the patient initiated knee straightening from a 15° starting angle at a rate of 2°/s until they perceived a change in knee angle. The test result was determined by calculating the average time for three consecutive measurements and multiplying this by 0.5. This value demonstrated an inverse relationship with the therapeutic effect. In the passive angle regeneration test, two angles in the ranges 80–100°, 40–60° and 0–20° were randomly selected for each test. The average of six measurements was calculated, with a higher average indicating a poorer treatment outcome.

#### Recovery of knee joint function

The recovery of knee joint function was assessed by knee Lysholm score and knee International Knee Documentation Committee (IKDC) function score before surgery, and at 3, 6 and 12 months after ACLR. The range for the total Lysholm score was 0–100, including squatting, pain, swelling, stair climbing, instability, need for support, limping and catching. The higher the score, the better the recovery of knee joint function. The total IKDC score ranged from 0 to 100, and included assessment items such as symptoms, function, daily activities and physical activities. The higher the score, the better the recovery of knee function.

#### Postoperative complications

Postoperative complications were recorded.

### Statistical analysis

Statistical analysis was performed using SPSS 26.0 software. Qualitative data were described by [*n* (%)] and a chi-squared was performed. Normally distributed quantitative data were described by mean standard deviation ($\bar{x}$ *± s*) for *t*-test, and skewed quantitative data were described by median and interquartile range [M (P25, P75)] for Mann–Whitney *U* test. A *p*-value <0.05 was considered statistically significant.

## Results

### Pain and swelling

The difference in pain and swelling between the two groups before reconstruction was not significant (*p* > 0.05). After reconstruction, pain and swelling were reduced in both groups, and the improvement was more obvious in the observation group (*p* < 0.05) (Table 2).

**Table 2:** Comparison of pain and swelling levels between the two groups (*n*=60)

Items	Observation group	Control group	*p*-value
Before reconstruction			
Pain (points)	6.00 (6.00, 7.00)	6.00 (5.00, 7.00)	0.086
Swelling (cm)	5.76 ± 0.68	5.70 ± 0.73	0.659
After reconstruction			
Pain (points)	1.00 (1.00, 2.00)^a^	2.00 (1.00, 2.00)^a^	**<0.001**
Swelling (cm)	1.61 ± 0.32^b^	2.73 ± 0.42^b^	**<0.001**

Note: ^a^*p* < 0.05 compared with pain in the same group before reconstruction.

^b^*p* < 0.05 compared with swelling in the same group before reconstruction.Normally distributed quantitative data were described by mean standard deviation ($\bar{x}$ *± s*) for *t*-test, and skewed quantitative data were described by median and interquartile range [M (P25, P75)] for Mann–Whitney *U* test.

### Knee range of motion

The difference in the ROM of knee flexion, extension, internal rotation and external rotation between the two groups before reconstruction was not significant (*p* > 0.05). The ROM increased in both groups after reconstruction, with the increase being greater in the observation group (*p* < 0.05) (Table 3).

**Table 3:** Comparison of knee range of motion between the two groups (*n* = 60)

Items	Observation group	Control group	*p*-value
Before reconstruction			
Flexion (°)	53.50 (46.25, 60.00)	52.50 (46.00, 59.00)	0.506
Internal rotation (°)	12.00 (9.00, 15.00)	12.00 (9.00, 14.00)	0.860
External rotation (°)	12.00 (8.00, 16.00)	13.00 (9.00, 17.00)	0.280
After reconstruction			
Flexion (°)	120.50 (116.25, 124.75)^a^	102.00 (96.00, 108.00)^a^	**<0.001**
Internal rotation (°)	27.50 (26.00, 29.00)^b^	23.00 (20.25, 25.00)^b^	**<0.001**
External rotation (°)	36.00 (33.00, 39.00)^c^	30.00 (26.00, 34.00)^c^	**<0.001**

Note: ^a^*p* < 0.05 compared with flexion and extension in the same group before reconstruction.

^b^*p* < 0.05 compared with internal rotation in the same group before reconstruction.

^c^*p* < 0.05 compared with external rotation in the same group before reconstruction.Skewed quantitative data were described by median and interquartile range [M (P25, P75)] for Mann–Whitney *U* test.

### Proprioception

The difference in knee joint proprioception between the two groups before reconstruction was not significant (*p* > 0.05). The TTDPM and passive angle regeneration test results for the two groups at 3, 6 and 12 months after reconstruction were lower, and the observation group had lower results (*p* < 0.05) (Table 4).

**Table 4:** Comparison of proprioception between the two groups (*n* = 60)

Items	Observation group	Control group	*p*-value
Before reconstruction			
Threshold to detection of passive motion (°)	3.40 (3.03, 3.70)	3.30 (3.03, 3.80)	0.827
Passive angle regeneration test results (°)	6.70 (5.30, 8.35)	6.75 (5.60, 8.00)	0.807
3 months after reconstruction			
Threshold to detection of passive motion (°)	2.40 (2.13, 2.70)^a^	2.90 (2.53, 3.20)^a^	**<0.001**
Passive angle regeneration test results (°)	5.25 (4.70, 5.80)^b^	6.15 (4.70, 6.70)^b^	**0.001**
6 months after reconstruction			
Threshold to detection of passive motion (°)	2.10 (1.80, 2.30)^a^	2.30 (2.10, 2.50)^a^	**<** **0.001**
Passive angle regeneration test results (°)	3.65 (3.23, 4.10)^b^	4.05 (3.60, 4.50)^b^	**<** **0.001**
12 months after reconstruction			
Threshold to detection of passive motion (°)	1.10 (1.00, 1.20)^a^	1.10 (1.00, 1.20)^a^	0.806
Passive angle regeneration test results (°)	2.90 (2.53, 3.30)^b^	2.95 (2.60, 3.30)^b^	0.495

Note: ^a^*p* < 0.05 compared with threshold to detection of passive motions in the same group before reconstruction.

^b^*p* < 0.05 compared with passive angle regeneration test results in the same group before reconstruction.Skewed quantitative data were described by median and interquartile range [M (P25, P75)] for Mann–Whitney *U* test.

### Knee joint function

The preoperative knee function scores of the two groups did not differ significantly (*p* > 0.05). The Lysholm and IKDC function scores of the two groups were increased at 3, 6 and 12 months postoperatively, and were higher for the observation group than for the control group at 3 and 6 months postoperatively (*p* < 0.05) (Table 5).

**Table 5:** Comparison of knee function between the two groups (*n* = 60)

Items	Observation group	Control group	*p*-value
Before reconstruction			
Lysholm score (points)	49.50 (44.00, 56.00)	48.50 (43.25, 54.00)	0.281
IKDC score (points)	41.50 (37.00, 46.00)	42.50 (37.00, 48.00)	0.427
3 months after reconstruction			
Lysholm score (points)	79.00 (73.25, 84.00)^a^	62.00 (56.00, 68.00)^a^	**<** **0.001**
IKDC functionality score (points)	85.00 (80.00, 90.00)^b^	79.00 (72.00, 85.00)^b^	**<** **0.001**
6 months after reconstruction			
Lysholm score (points)	88.00 (86.00, 90.00)^a^	77.00 (73.00, 80.00)^a^	**<** **0.001**
IKDC score (points)	91.00 (84.00, 96.00)^b^	88.00 (81.25, 92.75)^b^	0.029
12 months after reconstruction			
Lysholm score (points)	95.00 (93.00, 97.00)^a^	94.00 (92.00, 96.00)^a^	0.196
IKDC score (points)	92.00 (85.25, 97.00)^b^	91.50 (85.00, 95.75)^b^	0.274

Note: ^a^*p*<0.05 compared with Lysholm score in the same group before reconstruction.

^b^*p*<0.05 compared with IKDC function score in the same group before reconstruction.Skewed quantitative data were described by median and interquartile range [M (P25, P75)] for Mann–Whitney *U* test.

IKDC = International Knee Documentation Committee.

### Postoperative complications

The incidence of postoperative complications in the observation group was 13.33% (8 of 60), less than that in the control group 15.00% (9 of 60; *p* > 0.05) (Table 6). Qualitative data were described by [*n* (%)].

**Table 6:** Comparison of postoperative complications between the two groups (*n* = 60)

Items	Observation group, *n* (%)	Control group, *n* (%)	*p*-value
Postoperative complications	8 (13.33)	9 (15.00)	0.793
Swelling of soft tissue due internal haemorrhage	4 (6.67)	2 (3.33)	–
Infections	1 (1.67)	1 (1.67)	–
Quadriceps atrophy	3 (5.00)	4 (6.67)	–
Deep vein thrombosis	0 (0.00)	1 (1.67)	–
Rigid joint	0 (0.00)	1 (1.67)	–

## Discussion

In the treatment of ACL injuries, ACLR remains the gold standard. Postoperative rehabilitation improves surgical outcomes and return-to-sport results significantly.^8,9^ Given that, this study, based on exercise rehabilitation therapy, performed stump-preserving or stump-eliminating ACLR and compared their efficacy and clinical effect on patients’ knee function recovery.

In the early postoperative rehabilitation stages following ACLR, it is vital to control pain and swelling. Diminishing pain and swelling enhances ROM and quadriceps activity, while lowering the likelihood of restricted ROM and contracture, potentially leading to future gait abnormalities and delay in advancing to subsequent stages.^10^ Pain and swelling were reduced in both groups after ACLR, and the improvement was more pronounced in the observation group. The ROM was evaluated for extension and flexion limitation. It was observed that the ROM of knee flexion, extension, internal rotation and external rotation increased in both groups after ACLR, and the improvement was more obvious in the observation group.

Stump-preserving ACL promotes the growth of proprioceptors, neurons and nerve-related gene expression, indicating enhancement of proprioception of the knee joints in the early stage.^11^ Stump-preserving ACLR provides better proprioception than standard ACLR and yields equivalent functional outcomes.^12,13^ This research detected that the TTDPM and passive angle regeneration test results at 3, 6 and 12 months after ACLR were reduced in both groups, and were lower in the observation group than in the control group. The reason for this is that there are a large number of mechanoreceptors in the ACL, and preserving the ACL stump at the tibial end facilitates the mechanoreceptors to grow into the tendon graft, ensures that the synovial membrane covers the surface of the graft well and promotes rapid recovery of knee joint function and proprioception. Furthermore, stump-preserving ACLR helps stabilise the ligament attachment of the bone in the early stage, and retaining the neuronal receptors of the ligament itself can enhance proprioception. With continuous improvement in the patient’s motor function, the ligament will further stabilise and grow in the later stage, further improving the degree of proprioception.

Lysholm and IKDC functional scores increased in both groups at 3, 6 and 12 months postoperatively and were higher in the observation group than in the control group at 3 and 6 months postoperatively. This result suggests that the implementation of ACLR therapy can positively treat patients with ACL injuries to a certain extent, but compared with stump-eliminating ACLR, stump-preserving ACLR is more effective in patients with ACL injuries, and it can effectively restore the patients’ sensory function and knee joint function. The reason for this is that stump-eliminating ACLR slows the process of vascular and neural growth because of the cleaning of the stump, which in turn slows the recovery of the knee function. Stump-preserving ACLR demonstrates a robust proprioceptive system that plays a crucial role in conveying both the static mechanical stability of the knee joint and the impact of nerve afferent effects. These distinctive nerve reflexes enhance the dynamic stability of the knee joint, thereby promoting healing of the tendon–bone complex in the later stages of recovery. Ultimately, these unique nerve reflexes contribute to the overall improvement in knee joint function.

Furthermore, although stump-preserving ACLR shows potential for improving graft healing by promoting cell proliferation, revascularisation and regeneration of proprioceptive organs in the reconstructed ACL, it is important to acknowledge that this approach is not without complications.^14^ In this study, the incidence of complications was lower in the observation group than in the control group, but the difference was not significant. This suggests that in the treatment of ACL injuries, the postoperative complications of arthroscopic stump-preserving reconstruction are comparable with those of stump-eliminating ACLR.

## Conclusion

Stump-preserving ACLR can promote proprioceptive recovery and improve knee function in patients with ACL injuries, which is superior to stump-eliminating ACLR. However, multicentre studies and a larger population size are required to verify the conclusions reached in this study.

## Funding

This study was supported by the Ankang Science and Technology Research and Development Program Project (No.AK2022-SF-020).
